# Addressing the growing burden of non–communicable disease by leveraging lessons from infectious disease management

**DOI:** 10.7189/jogh.06.010304

**Published:** 2016-06

**Authors:** Peter Piot, Aya Caldwell, Peter Lamptey, Moffat Nyrirenda, Sunil Mehra, Kathy Cahill, Ann Aerts

**Affiliations:** 1 London School of Hygiene and Tropical Medicine, London, UK; 2 Innovative Healthcare Delivery Solutions, Novartis Foundation, Basel, Switzerland; 3 Family Health International 360, Accra, Ghana; 4 Malawi Epidemiology and Intervention Research Unit, Malawi; 5 MAMTA Health Institute for Mother and Child, India; 6 PATH, Seattle, Washington, USA; 7 Novartis Foundation, Basel, Switzerland

In recent decades, low– and middle–income countries (LMICs) have achieved decreased morbidity and mortality associated with infectious diseases and poor maternal– and child–health (MCH). However, despite these advances, LMICs now face an additional burden with the inexorable rise of non–communicable diseases (NCDs).

Deaths due to NCDs in LMICs are expected to increase from 30.8 million in 2015 to 41.8 million by 2030 [1]. While improvements in life expectancy, lifestyle and urbanisation go some way to explaining why more people in LMICs are affected by NCDs, it is less clear why these populations are contracting NCDs at a younger age and with worse outcomes than in high–income countries (HICs) [2]. Despite having a lower cardiovascular disease risk factor burden, LMIC populations have a four–fold higher mortality rate from cardiovascular events than HIC populations [3] in part due to a lack of access to quality, integrated health services and the poor availability of early interventions and effective NCD prevention programmes. The HIV/AIDS epidemic was the last time the world confronted a global health challenge that so disproportionately caused premature adult deaths in LMICs.

The conclusion is unavoidable: the time to act is now. Prevention of NCDs at a population and an individual level is key and requires policy and structural changes. We have a unique opportunity to learn from the successes of infectious disease control programmes in LMICs and leverage these to address the growing NCD burden. Translatable learnings include: 1) emphasizing primary prevention, particularly in those at highest risk; 2) targeting service delivery to high–risk populations; 3) enabling access to adequate, affordable care at community level; 4) engendering patient empowerment and involving people affected by chronic conditions; 5) enabling access to quality drugs and adherence programmes; 6) regularly measuring the effectiveness and impact of programmes to ensure their appropriateness and improvement; and 7) creating an environment of health financing for universal coverage.

Innovations to counter the emerging NCD epidemic must encompass both prevention and the delivery of care. Infectious disease programmes have used task–shifting, where less skilled health workers and community members are involved in delivery of health services. In India, we have seen this used for NCDs in the Arogya Kiran model where the existing health workforce was overstretched. Volunteers and teachers successfully delivered diabetes and hypertension screening and management to over 600 000 people [4]. Patient empowerment, and community involvement in health care delivery and governance, will be critical in tackling NCDs, since most are chronic conditions, which initially present silently and require long–term management [5].

In Malawi, recognizing the close relationship of HIV infection and cardiovascular diseases has led to screening for hypertension being integrated into HIV care [6]. In Ghana, decentralised community–based hypertension care, using digital technology, is helping to empower patients to manage their own disease: a model that is again adapted from HIV management [7]. We are also starting to see examples in India of MCH care coupled with life–long NCD screening and awareness programmes [8].

While these examples of managing the dual burden of infectious diseases and NCDs are encouraging, more needs to be done. The largest gap is in NCD prevention. Tackling the obesity epidemic and wrestling with the issues around curbing tobacco sales and smoking are rightly high on the NCD prevention agenda. The greatest opportunity is preventing a tobacco–related epidemic in sub–Saharan Africa where smoking levels are still low. Health budgets and development assistance for health must allocate resources commensurate with the dual disease burden. Health spending of governments in LMICs has tripled over the past 20 years, but remains low [9]. In addition, more health care models should consider diversified revenue streams or hybrid financing (eg, tiered payment schemes) to ensure sustainability. If equity is to be improved, patients need access to quality health care, through sustainable health–financing systems for universal health coverage, while reducing out–of–pocket expenditure for the under–served population.

**Figure:**
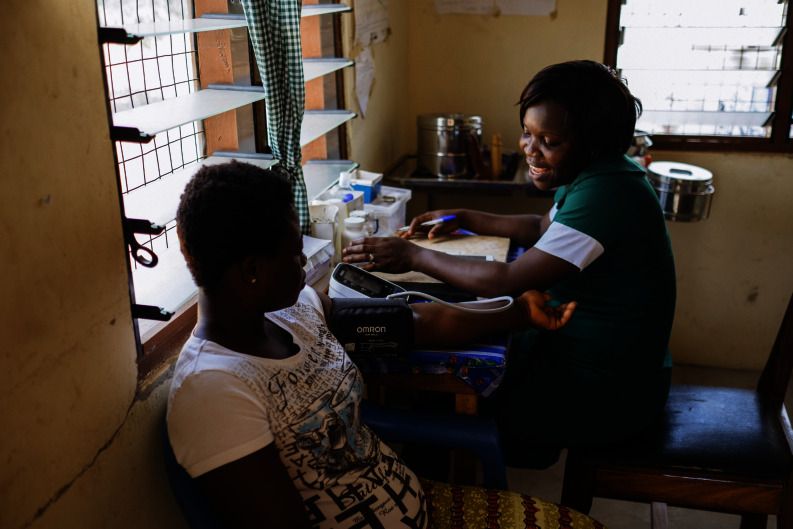
Photo: © Nana Kofi Acquah/Novartis Foundation

Implementing such models will require strong government leadership and interventions, and partnerships across the public and private sectors. Some public–private partnerships (PPPs) in infectious diseases have demonstrated their potential to catalyze the delivery of, and access to, prevention and care through providing complementary strengths [10]. The private sector draws on its business and scientific expertise, focusing on strong results–based operations, whereas the public sector brings a wealth of expertise in implementation with equity, management and documentation.

The end–users of the services, including patients and health care providers, also need to be included from the outset to ensure that the models are people–centered, co–created, adapted to prevailing contextual nuances, and sustainable. If we build on what we have learnt from infectious disease management, we could have a transformational impact on the growing NCD burden.
